# Immunotherapy in cervical cancer: From the view of scientometric analysis and clinical trials

**DOI:** 10.3389/fimmu.2023.1094437

**Published:** 2023-02-03

**Authors:** Yang Xing, Feroza Yasinjan, Yajie Du, Huayue Geng, Ying Zhang, Minghua He, Rui Guo, Lei Yang, Jiayue Cui, Dongmei Mu, Ziling Liu, Hong Wang

**Affiliations:** ^1^ Cancer Center, The First Hospital of Jilin University, Changchun, China; ^2^ College of Computer Science and Technology, Jilin University, Changchun, China; ^3^ Clinical Laboratory, The First Hospital of Jilin University, Changchun, China; ^4^ Department of Histology and Embryology, College of Basic Medical Sciences, Jilin University, Changchun, China; ^5^ Division of Clinical Research, The First Hospital of Jilin University, Changchun, China

**Keywords:** immunotherapy, cervical cancer, clinical trials, scientometric analysis, HPV

## Abstract

**Background:**

Cervical cancer is the fourth most cancer and the fourth leading cause of cancer-related deaths in women worldwide. Current treatment for patients with advanced cervical cancer is limited. And in the urgent demand for novel effective therapies both as the first and the second line treatment for these patients, immunotherapy is developing fast and has made some achievements.

**Methods:**

This study incorporated 1,255 topic-related articles and reviews from 1999 to 2022 in the Web of Science Core Collection (WoSCC). The WoS platform, Citespace, and VOS viewer provided the annual distribution of publications and citations, the analysis of researching countries and institutions, references, keywords (co-occurrence analysis, burst analysis, and timeline view analysis), and researching authors, respectively. For clinical trials, 720 trials and 114 trials from ClinicalTrials.gov and ICTRP were retrieved, respectively. And 296 trials were finally incorporated into the analysis.

**Results:**

The scientometric analysis showed that the study of immunotherapies in cervical cancer developed fast in recent years. Most publications were from the United States, followed by China. Seven of the top 10 co-cited references belong to clinical trials, and five of them were published in recent five years. There are lots of clinical trials us specific treatment patterns, some of which have represented excellent effects.

**Conclusions:**

Both the scientometric analysis of the 1,255 publications and the analysis of clinical trials showed that the field of immunotherapies in cervical cancer developed so fast in recent years. It was found that a lot of clinical trials using various immunotherapies (mainly vaccine therapy, adoptive cell therapy, immune checkpoint blockade, and antibody-drug conjugate) for advanced cervical cancer are currently ongoing or have represented considerable effect. Centered in immunotherapies, immune checkpoint blockades have represented great efficacy and huge potential, especially combined with other therapies such as chemotherapy, targeted therapy, and other immunotherapies.

## 1 Introduction

Cervical cancer is not only the fourth most common cancer but also the fourth cause of cancer deaths in women worldwide (1). And for women aged 20 to 39 years in the United States and women aged 15 to 44 years in China, cervical cancer is the second and third main cause of cancer deaths, respectively (2, 3). With the gradual application of cervical cancer screening such as cervical cytological examination (Pap test) and high-risk human papillomavirus testing, and preventive vaccinations, the incidence of cervical cancer has remarkably decreased in developed countries (4–6). However, many developing countries with fewer healthcare resources still have the highest incidence and mortality rates of cervical cancer (7). According to a worldwide analysis in 2018 (7), there were about 84% of cervical cancer cases and 88% of cervical cancer deaths occurred in low-resource countries worldwide.

The therapeutic regimen for cervical cancer is classified by the stages of the disease, which are determined by the International Federation of Gynecology and Obstetrics (FIGO) (8, 9) or the American Joint Committee on Cancer and International Union for Cancer Control tumor–node–metastasis (TNM) staging system (10). Current treatment for cervical cancer includes surgery, radiotherapy, chemoradiotherapy, and systemic therapy such as chemotherapy, immunotherapy, targeted therapy, and their reasonable combined regimens. And the standard treatments for different stages or classifications of cervical cancer are different. For patients with locally advanced cervical cancer (stage IIb–IVa), concurrent chemoradiotherapy has been set as the international standard regimen since 1999 (11). For recurrent or metastatic (stage IVb) cervical cancer, the standard care choice is the combination of cisplatin, paclitaxel, and Bevacizumab with an overall response rate (ORR) of 48% and an median overall survival (mOS) of 17 months (12). However, there are very few choices for the second-line and above treatment of these cervical cancer patients. In the urgent demand for novel effective therapies both in the first and the second line treatment in these patients with advanced cervical cancer, immunotherapy is developing fast and has made some achievements. There are many immunogenic characteristics for cervical cancer, such as tumor antigens induced by HPV, high TMB (tumor mutational burden), infiltration of immune cells (particularly CD8+ lymphocytes), and multiple checkpoint control targets, which support the feasibility of immunotherapy intervention (13, 14).

Some types of immunotherapies such as vaccine therapy and adoptive cell therapy (ACT) were experimented and applied in cervical cancer early. But their efficacy is usually not satisfactory. The successful application of immune checkpoint blockades (ICBs) in metastatic melanoma brought new hopes to cervical cancer (15, 16). Up to now, Pembrolizumab and Cadonilimab (AK104) have been approved by FDA and National Medical Products Administration (China) respectively, in the treatment of advanced cervical cancer (17), which demonstrated the fast development and great prospect of immunotherapies in cervical cancer.

Based on the fast development of immunotherapies in cervical cancer, a scientometric analysis of the related publications from WoS and an analysis of related clinical trials were made to reveal the trends and research foci of this field (18, 19). And some significant clinical trials with promising results were discussed.

## 2 Materials and methods

### 2.1 Scientometric analysis

Web of Science Core Collection (WoSCC) is the most fundamental data source for both Citespace (6.1.R3) and VOS viewer (1.6.18). And the source of this scientometric analysis was the Science Citation Index Expanded (20) in WoSCC. The retrieval terms were made based on the Medical Subject Headings (Mesh) database: TS = (Cervical Cancer OR Cervix Cancer) AND TS=(immunotherapy). The type of documents: Article and Review. The language: English. The publication years were from 1999 to 2022. The search process was conducted on September 25, 2022. Finally, the information from 1,255 documents was downloaded in the form of Plain Text File and Tab Delimited File, respectively.

Web of Science (WoS). WoS platform provided the distribution of publications and citations by year.

Citespace (6.1.R3). The 1,255 results were downloaded from WoSCC in the Plain Text File form. The process of removing duplicates was conducted. And there were no duplicate records. Statistics of fields were obtained: 1247 publications (99.36%) with abstracts, 1255 publications (100%) with DOI numbers, 1252 publications (99.76%) with author keywords, 1255 publications (100%) with subject categories, and 1254 publications (99.92%) with cited references. Before the analysis, Citespace was adjusted by: 1) Time Slicing: 1999 Jan - 2022 October; 2) Selection Criteria: Top 50 levels from each slice; 3) Functions of Pruning: Retain the default. In this study, Citespace was used to provide analyses of studying countries and institutions, references, and keywords (including co-occurrence analysis, burst analysis, and timeline view analysis).

VOS viewer (1.6.18). The 1,255 results were downloaded from WoSCC in the Tab Delimited File form. VOS viewer was used to make analyses of researching authors of this field.

### 2.2 Clinical trails

ClinicalTrials.gov (https://www.clinicaltrials.gov) and WHO ICTRP (https://trialsearch.who.int) were applied as the source of clinical trials in this study (21). The searching strategies in ClinicalTrials.gov/ICTRP: 1) Condition or Disease/Condition = (Cervical Cancer), 2) Other terms/Innervation = (Immunotherapy OR PD-1 OR PD-L1 OR CTLA-4 OR TIGIT OR LAG3 OR TIM-3 OR A2AR OR OX40 OR ICOS OR 4-1BB OR Cadonilimab OR AK-104 OR Camrelizumab OR Nivolumab OR Pembrolizumab OR Sintilimab OR Atezolizumab OR Ipilimumab OR Durvalumab OR Zalifrelimab OR Dostarlimab OR Balstilimab OR GEN1046 OR M7824 OR SHR-1701 OR Adoptive Cell Therapy OR TIL OR TCR-T OR CAR-T OR CIK OR LAK OR DC OR Lifileucel OR Antibody Drug Conjugate OR Tisotumab Vetodin OR TNF OR IFN OR Interleukin OR Therapeutic Vaccine OR Therapeutic Vaccination). There were 720 trials from ClinicalTrials.gov and 114 trials from ICTRP. After duplicating by Trial IDs and reviewing each trial by two independent authors, there were 296 clinical trials incorporated into this study. The time of searching and handling the data was October 2022. For the cancer stages of cervical cancer, the FIGO 2018 staging system for cervical cancer was adopted in this study (8, 22). 1Recurrent/Metastatic (stage IVB) and 2Locally Advanced (stages IB3 and IIA2-IVA) were the main classifications used to describe the stages of cervical cancer. In Microsoft Excel (2019), the specific and detailed information of 296 clinical trials was recognized by two independent authors, including Cancer Stages (r/m, LA, r/m+ LA, and others), Treatment Modes (ICB, ACT, ADC, Vaccines, and so on), Supplement Information (drug targets, detailed therapies, and so on), Phases (phase I, phase I/II, phase II, phase II/III, and phase III), and Status (Recruiting, Not Recruiting, Terminated, and so on). After the statistical process of all clinical trials, the corresponding analyses were made.

## 3 Results

### 3.1 Scientometric analysis

#### 3.1.1 Distribution of publications and citations by year

**Figure 1:**
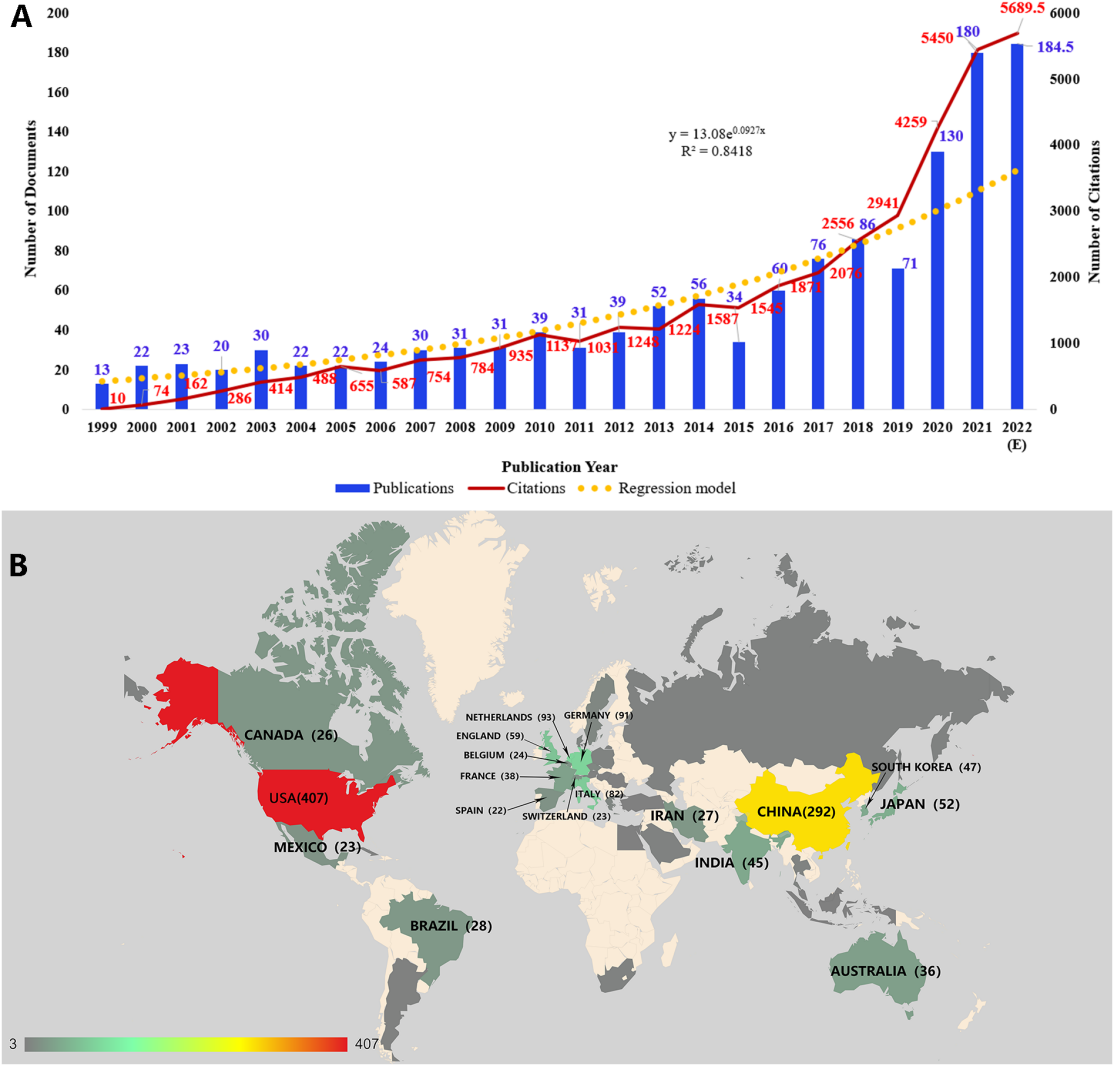
**(A)** The distribution of publications and citations by year. The regression formula is y = 13.08e0.0927x, R2 = 0.8418. Since the publications in our current study did not cover all publications in 2022, the expected number of publications in 2022 was calculated. **(B)** The geographical visualization of publications for immunotherapy in cervical cancer.

According to the specific searching strategy in WoSCC, a total of 1,255 documents were included in our study. There were 35,867 citations for all the documents, and an average of 28.58 citations per document were noted. The H-index was 85, indicating that 85 documents have obtained more than 85 citations. **Figure 1A** shows the distributions of both publications and citations by year. And it also represented an increasing growth trend from 1999-2022. Only 13 publications were published in 1999, and the annual publication output maintained a relatively low level in the next few years. During the decade from 2009 to 2018, the publication number increased slowly. And the next few years after 2018 witnessed a sharp rise in both publication and citation numbers. To know how publications in this field changes with time clearly, we adopted a regression model y = 13.08e^0.0927x^ (R^2^ = 0.8418) in the graph.

#### 3.1.2 Related countries (or regions) and institutions of this field

There were 22 countries or regions with ten or more publications in the field of immunotherapy in cervical cancer (**Figure 1B**). The United States had the most publications (n=407), accounting for 25.91% of all. China ranked second with 292 publications (18.59%). And publications of these two countries were much more than the following countries such as the Netherlands (n=93, 5.92%), Germany (n=91, 5.79%), and Italy (n=82, 5.22%).

**Figure 2:**
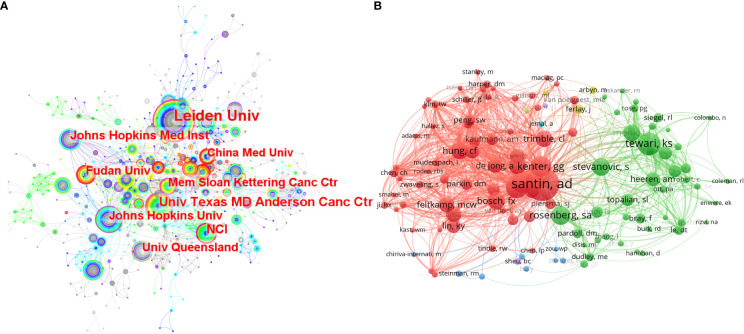
**(A)** The co-occurrence map of the researching institutions. The size of a node lies on the publication number of an institution. The width of the purple circle around a node represents the betweenness centrality of an institution. **(B)** The network map of researching authors who were co-cited over 40 times.

The research institutions were also analyzed in Citespace (**Figure 2A**). A total of 930 institutions had related publications in this field. Leiden University ranked first with the highest number of publications (n=53). There were six of the top 10 institutions with the highest publications from the United States. And the proportion was the same for the top 10 institutions with the highest betweenness centralities. Betweenness centralities can represent the importance of a node in the visualization map (18). And institutions with higher centralities mean that they are more predominant than others in this field. In this analysis, the five institutions with centralities over 0.10 were the National Cancer Institute (the United States) (0.16), Leiden University (Netherlands) (0.15), Johns Hopkins University (the United States) (0.14), University of Texas MD Anderson Cancer Center (the United States) (0.14), and Memorial Sloan-Kettering Cancer Center (the United States) (0.10).

#### 3.1.3 Authors

We used VOS viewer to analyze the publications, citations, and co-citations of authors in this field. There were 11 highly published authors who had more than ten publications related to immunotherapy in cervical cancer. And van der Burg SH ranked first with 30 publications, followed by Hung Chien-Fu (n=22), Cornelis JM Melief (n=18), and Kenter GG (n=17). For highly cited authors in this field, van der Burg Sjoerd H also had the highest citations (n=2,303), followed by Cornelis J.M. Melief (n=2,167). Co-citation analysis can represent the influence of an author in a way that is more concentrated on the designated topic than citation analysis (23). Through VOS viewer, 122 authors who had been co-cited at least 40 times were incorporated to make the network of related authors (**Figure 2B**). The authors who have the same color were in the mutual cluster, meaning they were co-cited very often. Further, some active co-citation relationships can be observed, such as Santin AD and Kenter GG, Tewari KS, and Stevanovic Sanja. Among all the highly co-cited authors, there were 24 authors who were co-cited over 100 times. Santin AD ranked first with 279 co-citations, followed by Tewari KS (n=188) and van der Burg SH (n=179).

#### References

3.1.4

**Figure 3:**
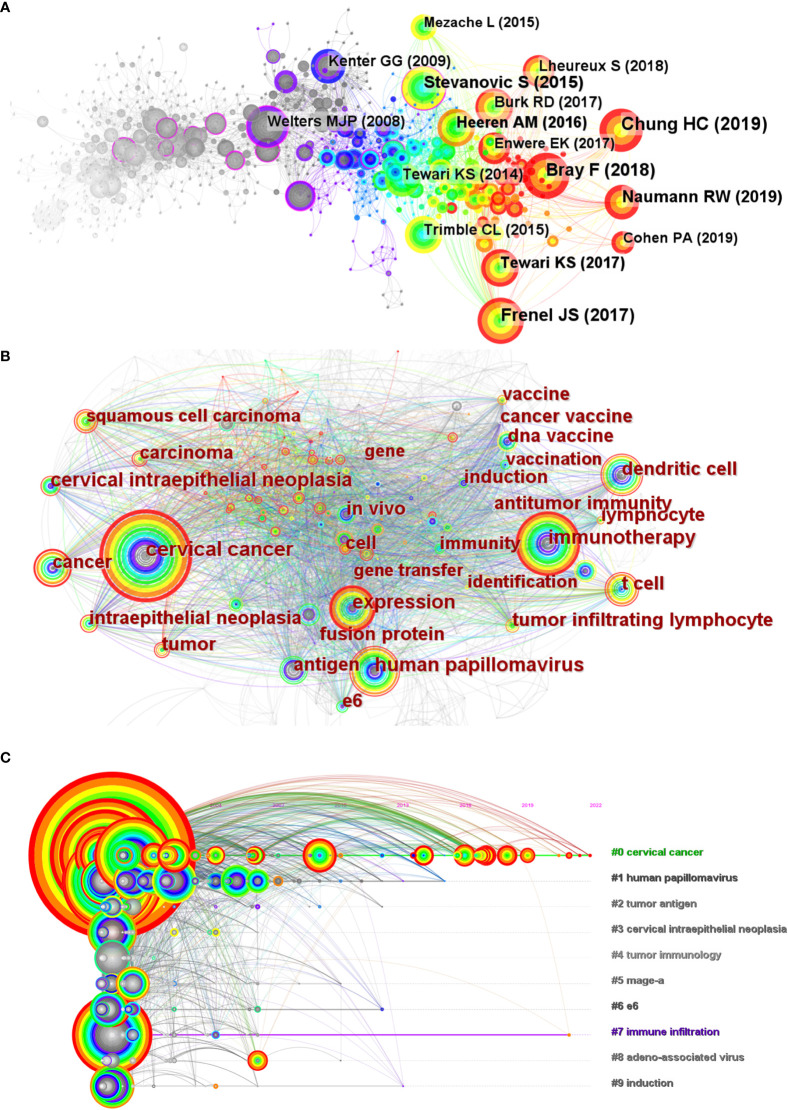
**(A)** The co-citation analysis of references. The highly co-cited references were arranged by year. **(B)** The co-occurrence map of the keywords with high frequency. The three basic classifications were revealed, including cervical cancer, HPV, and immunotherapy. **(C)**The timeline view of keywords from Citespace. The leading ten clusters were selected in the analysis. The algorithm of showing labels of clusters was LLR.

The development and evolution of research on immunotherapy in cervical cancer can be explored through the co-citation analysis of the references (**Figure 3A**). As shown in the figure, more than half of the highly co-cited references appeared in recent five years, indicating that the field has developed so fast recently. The ten most co-cited articles were selected for further analysis (**Table 1**). There were seven clinical trials on the list, in which three trials were associated with ICBs such as Pembrolizumab and Nivolumab, two trials were about vaccine therapies, one trial was about tumor-infiltrating T cells, and one trial was associated with Bevacizumab. As the highest co-cited references in this analysis, Pembrolizumab and its phase II KEYNOTE-158 study started the formal use of ICBs in cervical cancer. In June 2018 Pembrolizumab was approved by the FDA to treat advanced cervical cancer (PD-L1 positive) in the second line. And in October 2021 Pembrolizumab combined with chemotherapy was approved as the first-line treatment of persistent, recurrent, or metastatic cervical cancer based on its KEYNOTE-826 study. As another classical PD-1 inhibitor, Nivolumab has also revealed good effects on advanced cervical cancer. The CheckMate 358 trial studied Nivolumab both as monotherapy (28) and combined with ipilimumab (33) in recurrent or metastatic cervical cancer. In addition, it is worth noting that two trials related to vaccine therapies were conducted in 2008 and 2009, much earlier than the other five trials. Bevacizumab is one of the few monoclonal antibodies (anti-VEGF) used in the treatment of advanced cervical cancer. In 2014, Bevacizumab was approved by the FDA to combine with chemotherapy for the treatment of metastatic, persistent, or recurrent cervical cancer based on the consequences of the phase III GOG-240 study (29). And Bevacizumab also plays as a common option when some new drugs are combined with standard chemotherapy in clinical trials. Besides the review of the report on the global burden of cancer, the remaining two references were also related to immunotherapies in cervical cancer. Heeren AM et al. (30) researched the clinical importance of PD-L1 in cervical cancer, and Burk RD et al. (32) made a comprehensive genomic analysis of cervical cancer in which the amplifications in PD-L1/PD-L2 were represented.

**Table 1:** The top 10 co-cited documents.

Year	Title	Type	First author	Journal	Focus and main idea	IF (2022)	JCR	Co-citation
2019	Efficacy and Safety of Pembrolizumab in Previously Treated Advanced Cervical Cancer: Results from the Phase II KEYNOTE-158 Study	Clinical Trial	Chung HC (24)	J CLIN ONCOL	A phase II basket study investigated the antitumor activity and safety of pembrolizumab in advanced cervical cancer.	50.717	Q1	93
2017	Safety and Efficacy of Pembrolizumab in Advanced, Programmed Death Ligand 1-Positive Cervical Cancer: Results from the Phase Ib KEYNOTE-028 Trial	Clinical Trial	Frenel JS (25)	J CLIN ONCOL	A phase Ib trial suggested that pembrolizumab is well tolerated and has durable antitumor activity in PD-L1–positive advanced cervical cancer.	50.717	Q1	80
2018	Global cancer statistics 2018: GLOBOCAN estimates of incidence and mortality worldwide for 36 cancers in 185 countries	Review	Bray F (26)	CA-CANCER J CLIN	A status report on the global burden of cancer.	286.130	Q1	78
2015	Complete regression of metastatic cervical cancer after treatment with human papillomavirus-targeted tumor-infiltrating T cells	Clinical Trial	Stevanovic S (27)	J CLIN ONCOL	A study investigated whether ACT could mediate regression of metastatic cervical cancer.	50.717	Q1	62
2019	Safety and Efficacy of Nivolumab Monotherapy in Recurrent or Metastatic Cervical, Vaginal, or Vulvar Carcinoma: Results from the Phase I/II CheckMate 358 Trial	Clinical Trial	Naumann RW (28)	J CLIN ONCOL	A phase I/II trial of nivolumab investigated the safety and efficacy of Nivolumab in recurrent/metastatic cervical and vaginal or vulvar cancers.	50.717	Q1	56
2017	Bevacizumab for advanced cervical cancer: Final overall survival and adverse event analysis of a randomized, controlled, open-label, phase 3 trial (Gynecologic Oncology Group 240)	Clinical Trial	Tewari KS (29)	LANCET	A phase III randomized trial investigated that chemotherapy with bevacizumab could improve OS in advanced cervical cancer.	202.731	Q1	46
2016	Prognostic effect of different PD-L1 expression patterns in squamous cell carcinoma and adenocarcinoma of the cervix	Article	Heeren AM (30)	MODERN PATHOL	Clinical significance of PD-L1 expression in cervical cancer.	8.209	Q1	45
2008	Induction of tumor-specific CD4+ and CD8+ T-cell immunity in cervical cancer patients by a human papillomavirus type 16 E6 and E7 long peptides vaccine	Clinical Trial	Welters MJP (31)	CLIN CANCER RES	A study evaluated the effect of HPV16 E6 and E7 synthetic long peptides vaccine on the antigen-specific T-cell response in cervical cancer patients.	13.801	Q1	44
2017	Integrated genomic and molecular characterization of cervical cancer	Article	Burk RD (32)	Nature	The largest comprehensive genomic study of cervical cancer to date.	69.504	Q1	44
2009	Vaccination against HPV-16 oncoproteins for vulvar intraepithelial neoplasia	Clinical Trial	Kenter GG	NEW ENGL J MED	Immunogenicity and efficacy of a synthetic long-peptide vaccine in women with HPV-16–positive high-grade vulvar intraepithelial neoplasia.	176.079	Q1	42

#### 3.1.5 Co-occurrence analysis of keywords

Keywords can be regarded as highly condensed research topics. To discover the core content and research hotspots of this field, we used Citespace to make the keyword co-occurrence map (**Figure 3B**) which displayed the relationship between keywords. Further, there are three classifications of keywords, which were associated with (1) human papillomavirus (HPV) (2), cervical cancer, and (12) immunity and immunotherapy, respectively. And these three classifications form a triangle relationship in the figure. The six keywords with the highest frequency are cervical cancer (n=555), immunotherapy (n=288), the human papillomavirus (n=225), expression (n=208), dendritic cell (n=165), and t cell (n=156). In general, it reflects the core topic in our study, namely immunotherapies in cervical cancer caused by HPV.

#### 3.1.6 Timeline view of keywords

Through the cluster analysis of keywords, the different clusters were obtained along with the central keywords. The rank or the number of clusters is dependent on their sizes. The first #0 has the largest size, and the larger number of clusters means the smaller sizes. In this study, there were ten clusters selected to conduct the timeline view analysis, #0 cervical cancer, #1 human papillomavirus, #2 tumor antigen, #3 cervical intraepithelial neoplasia, #4 tumor immunology, #5 mage-a, #6 e6, #7 immune infiltration, #8 adeno-associated virus, and #9 induction. Based on the clustering analysis, the timeline view of keywords was made in the Citespace (**Figure 3C**).

It showed the development and the research hotspots of the topic field (immunotherapy in cervical cancer). And the figure represented the same three classifications as the co-occurrence map (1): cervical cancer (#0, #2, #3, #5) (2); human papillomavirus (#1, #6) (12); immunity and immunotherapy (#4, #7, #8, #9). Besides, it can also be noticed that cluster #7 tumor infiltration has continued to be active in recent years, indicating immunity and immunotherapy have received continuous attention.

#### 3.1.7 Burst analysis of keywords

**Figure 4:**
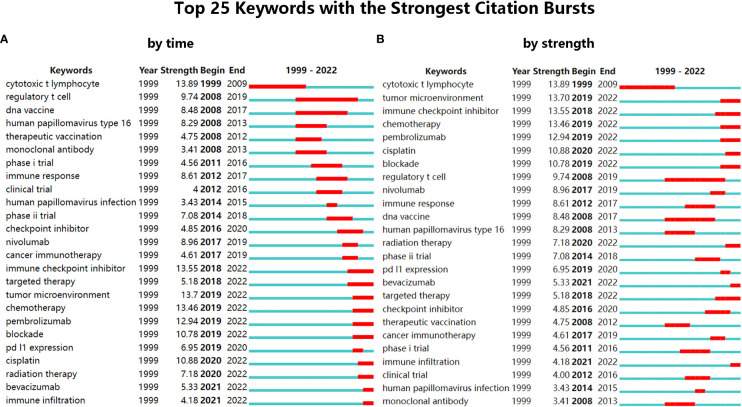
**(A)** The top 25 keywords with the strongest citation bursts, sorted by time. **(B)** The top 25 keywords with the strongest citation bursts, sorted by strength.

The burst detection in CiteSpace is based on Kleinberg’s algorithm (34). And through the burst analysis of keywords, the rapidly growing keywords in a short period will be obtained, which are also called burst keywords. Burst keywords mean the keywords have attracted an extraordinary degree of attention from the scientific community, which can help analyze the research trends of a field (18). The most representative 25 burst keywords were selected from the 99 burst keywords after burst analysis of keywords. And the keywords were sorted by time and by strength, respectively (**Figure 4A, B**). The strongest one is the cytotoxic T lymphocyte (13.89), which appeared early (1999) and lasted for a long time (11 years). Through further analysis, the 25 burst keywords can be summarized into two aspects: one is cervical cancer and its main cause (HPV), and the other is the treatment of cervical cancer (mainly immunotherapy). In recent years, there have been a lot of new burst keywords, indicating that the research topic of this field is expanding and updating quickly. And the three burst keywords that were concerned with clinical trials (phase I trial, clinical trial, and phase II trial) also indicated the active attempts in the field of immunotherapies for cervical cancer. In the past five years, the main burst keywords related to immunotherapy for cervical cancer included Nivolumab (8.96), cancer immunotherapy (4.61), immune checkpoint inhibitor (13.55), tumor microenvironment (13.7), Pembrolizumab (12.94), PD-L1 expression (6.95), and immune infiltration (4.18). And it showed that the research of ICBs such as Nivolumab and Pembrolizumab and their related fields were the main topics in recent years. Besides, the result of burst analysis is also consistent with the analysis of references that both analyses emphasized Pembrolizumab and Nivolumab.

### 3.2 Clinical trials

#### 3.2.1 Development and current status of related clinical trials

**Figure 5:**
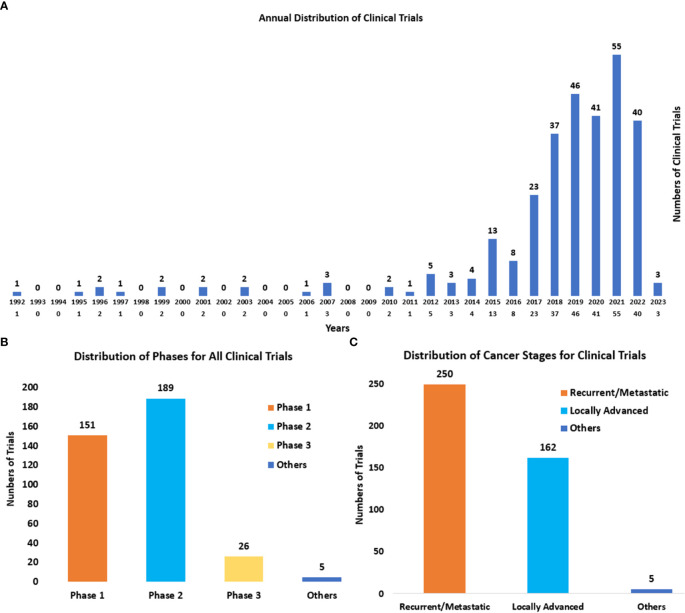
**(A)** The annual distribution of clinical trials from 1992 to 2023. The start date was regarded as the time of clinical trials. **(B)** The distribution of phases for all clinical trials. Others refer to the trials without known phases. **(C)** The distribution of cancer stages (recurrent/metastatic and locally advanced) for cervical cancer. Others mainly refer to early cervical cancers (stages IA1-IB2 and IIA1).

Through the analysis of clinical trials, it was found that the attempts of immunotherapies in cervical cancer started in 1992 (a clinical trial using Isotretinoin plus Interferon to treat patients with recurrent cancer (NCT00002506)). During the next 25 years, the development of immunotherapies for cervical cancer kept a slow pace. And over the past five years, lots of changes have occurred since the remarkable effect of immune checkpoint blockades on cervical cancer was represented. Both the scientometric analysis (**Figure 1A**) and the annual distribution of clinical trials (**Figure 5A**) showed the sharp development of immunotherapy in cervical cancer recently. The number of clinical trials that started in 2021 reached the peak (n=55). And a higher number of clinical trials can be expected in the future.

The phases of clinical trials (**Figure 5B**) were mainly Phase II (n=187) and Phase I (n=149). And it also displayed the wide exploration of novel immunotherapies in cervical cancer recently. There were also 26 clinical trials of Phase III, which represented the leading immunotherapies in the field of cervical cancer. And they are summarized in **Table 2**.

**Table 2:** All the related clinical trials in Phase III (or Phase II/III).

No.	Trial ID	Cancer Stage	Start Date	Result	Title
1	NCT03635567 (EUCTR2018-001440-53-DE)	r/m	Oct, 2018	Has Result	Efficacy and Safety Study of First-line Treatment With Pembrolizumab (MK-3475) Plus Chemotherapy Versus Placebo Plus Chemotherapy in Women With Persistent, Recurrent, or Metastatic Cervical Cancer (MK-3475-826/KEYNOTE-826) (35)
2	NCT03257267	r/m	Sep, 2017	Has Result	Study of Cemiplimab in Adults With Cervical Cancer (36)
3	NCT03556839	r/m	Sep, 2018	Active, not recruiting	Platinum Chemotherapy Plus Paclitaxel With Bevacizumab and Atezolizumab in Metastatic Carcinoma of the Cervix (37)
4	NCT03755739*	r/m+LA	Nov, 2018	no	Trans-Artery/Intra-Tumor Infusion of Checkpoint Inhibitors for Immunotherapy of Advanced Solid Tumors
5	NCT03830866 (EUCTR2018-002872-42-PL, EUCTR2018-002872-42-HU)	LA	Feb, 2019	no	Study of Durvalumab With Chemoradiotherapy for Women With Locally Advanced Cervical Cancer (CALLA) (38)
6	NCT03912415	r/m	Oct, 2019	no	Efficacy and Safety of BCD-100 (Anti-PD-1) in Combination With Platinum-Based Chemotherapy With and Without Bevacizumab as First-Line Treatment of Subjects With Advanced Cervical Cancer (FERMATA)
7	NCT03946358	r/m+LA	Feb, 2020	no	Combination of UCPVax Vaccine and Atezolizumab for the Treatment of Human Papillomavirus Positive Cancers (VolATIL)
8	NCT04157985	r/m+LA	Nov, 2019	no	Evaluating Length of Treatment With PD-1/PD-L1 Inhibitor in Advanced Solid Tumors
9	NCT04300647*	r/m	Jun, 2020	no	A Study of Tiragolumab Plus Atezolizumab and Atezolizumab Monotherapy in Participants With Metastatic and/or Recurrent PD-L1-Positive Cervical Cancer
10	NCT04697628	r/m	Feb, 2021	no	Tisotumab Vedotin vs Chemotherapy in Recurrent or Metastatic Cervical Cancer
11	NCT04806945	r/m	Sep, 2022	Withdrawn	A Phase III Study to Evaluate Efficacy and Safety of First-Line Treatment With HLX10 + Chemotherapy in Patients With Advanced Cervical Cancer
12	NCT04864782*	r/m	Sep, 2020	no	QL1604 Plus Chemotherapy Versus Chemotherapy in Subjects With Stage??, Recurrent, or Metastatic Cervical Cancer
13	NCT04906993	r/m	Jul, 2021	no	Camrelizumab Combined With Famitinib Malate for Treatment of Recurrent/Metastatic Cervical Cancer
14	NCT04943627 (EUCTR2021-002193-63-LT)	r/m	Aug, 2021	Withdrawn	Balstilimab Versus Investigator Choice Chemotherapy in Patients With Recurrent Cervical Cancer (BRAVA)
15	NCT04982237	r/m	Aug, 2021	no	A Study of AK104 Plus Platinum-containing Chemotherapy ± Bevacizumab as First-line Treatment for Persistent, Recurrent, or Metastatic Cervical Cancer
16	NCT05173272	LA	Sep, 2022	no	Induction Chemotherapy Combined With Immunotherapy Followed by Concurrent Chemoradiation in Advanced Cervical Cancer
17	NCT05179239	r/m	Feb, 2022	no	A Study of SHR-1701 Plus Platinum-containing Chemotherapy With or Without BP102 (Bevacizumab) as First-line Treatment in Cervical Cancer
18	NCT05234905	r/m	Mar, 2022	no	H101 Combined With Camrelizumab for Recurrent Cervical Cancer
19	NCT05235516	LA	Jun, 2022	no	A Study of AK104/Placebo Combined With Chemoradiotherapy For The Treatment of Locally Advanced Cervical Cancer
20	JPRN-JapicCTI-184042	r/m	Nov, 2018	no	Study of REGN2810 in Adults With Cervical Cancer
21	EUCTR2021-000179-36-ES	r/m+LA	Aug, 2021	no	An Open-label, Multicenter Follow-up Study to Collect Long-term Data on Participants from Multiple Bintrafusp alfa (M7824) Clinical Studies - Bintrafusp alfa Program Rollover Study
22	EUCTR2021-000179-36-IT	r/m+LA	Sep, 2021	no	An Open-label, Multicenter Follow-up Study to Collect Long-term Data on Participants from Multiple Bintrafusp alfa (M7824) Clinical Studies

r/m means recurrent or metastatic, and LA means locally advanced.

* means clinical trials are Phase II|Phase III.

The cancer stages for cervical cancer in clinical trials (**Figure 5C**) were mainly recurrent/metastatic (n=246), and locally advanced (n=159). The distribution also indicated the urgent need for more novel and effective therapies for these advanced cervical cancers.

#### 3.2.2 Immunotherapy strategies of related clinical trials

**Figure 6:**
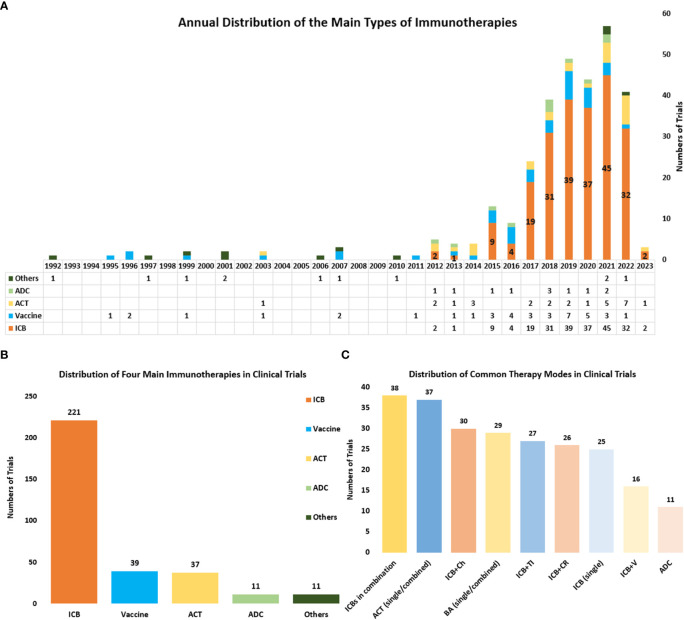
**(A)** The annual distribution of the main types of immunotherapies including ICBs, Vaccines, ACT, and ADC) from 1992 to 2023. Other therapies include cytokine therapy, oncolytic virus and so on, and these therapies were counted only when they were used as the main therapy. **(B)** The distribution of the main types of immunotherapies in all clinical trials. **(C)** The distribution of some common combined therapies. ICBs in combination referred to several ICBs that were used simultaneously in a clinical trial. ACT (single/combined) referred to ACT that was used as a single agent or combined with other therapies. BA (single/combined) referred to bispecific antibodies that were used as a single agent or combined with other therapies ICB plus Ch referred to ICB combined with chemotherapy. ICB plus CR referred to ICB combined with chemoradiotherapy. ICB plus TI referred to ICB combined with targeted therapy. ICB plus V referred to ICB combined with vaccine therapy.

Currently, there are four main kinds of immunotherapies in the treatment of cervical cancer, including ICBs, Vaccines, ACT, and antibody-drug conjugate (ADC). And the distribution and annual distribution of immunotherapies (**Figure 6A, B**) showed that ICBs have been the mainly researched immunotherapy in cervical cancer in recent years, with the highest frequency (n=195) in all clinical trials. ACT and ADC have also been gradually applied in cervical cancer recently. Differently, vaccine therapies which were first used in 1995, have a much longer history . And it has obtained continuous attention until now. In recent years the higher number of clinical trials related to vaccine therapies has also indicated its significance in the field of cervical cancer.

Through further analysis of clinical trials, we also obtained the distribution of nine common immunotherapies either as a single agent or combined with other regimens (**Figure 6C**). The combination of several ICBs is the commonest combined regimen (n=38). And the bispecific antibody is also getting popular and serves as a common immunotherapy in cervical cancer (n=27). ICB combined with chemotherapy (n=30) and ICB combined with chemoradiotherapy (n=26) are the commonly combined therapies that use both immunotherapy and traditional therapy. And the combined immunotherapies such as ICBs plus vaccine therapy (n=16) and ICBs plus ACT, are also widely researched.

#### 3.2.3 Clinical trials of some important immunotherapeutic drugs

Based on the current status of immunotherapies in cervical cancer, clinical trials of some important immunotherapeutic drugs were summarized and analyzed (**Table 3**, **S1**).

**Table 3:** The significant bispecific antibodies and their correspondingly selected clinical trials.

Drugs	No.	Has results	Trial ID	Phase	Cancer Stage	Treatment Mode	Title
AK104 PD-1 and CTLA-4	1	no	NCT05227651	Phase 2	LA	BA	AK104 in Neoadjuvant Treatment of Cervical Cancer
	2	yes	NCT04868708	Phase 2	r/m	BA+Ch+TI	A Study of AK104 (an Anti-PD-1 and Anti-CTLA-4 Bispecific Antibody) in Recurrent or Metastatic Cervical Cancer
	3	yes	NCT04380805	Phase 2	r/m	BA	A Study of AK104, a PD-1/CTLA-4 Bispecific Antibody in Subjects With Recurrent/Metastatic Cervical Cancer
	4	no	NCT05235516	Phase 3	LA	BA+CR	A Study of AK104/Placebo Combined With Chemoradiotherapy For The Treatment of Locally Advanced Cervical Cancer
	5	no	NCT04982237	Phase 3	r/m	BA+Ch+TI	A Study of AK104 Plus Platinum-containing Chemotherapy ± Bevacizumab as First-line Treatment for Persistent, Recurrent, or Metastatic Cervical Cancer
Bintrafusp Alfa PD-L1 and TGF-β	6	yes	NCT04432597	Phase 1|Phase 2	r/m+LA	BA+V	HPV Vaccine PRGN-2009 Alone or in Combination With Anti-PDL1/TGF-Beta Trap (M7824) in Subjects With HPV Associated Cancers
	7	yes	NCT03427411	Phase 2	r/m+LA	BA	M7824 in Subjects With HPV Associated Malignancies
	8	no	EUCTR2021-000179-36-ES EUCTR2021-000179-36-IT	Phase 3	r/m+LA	BA	An Open-label, Multicenter Follow-up Study to Collect Long-term Data on Participants from Multiple Bintrafusp alfa (M7824) Clinical Studies - Bintrafusp alfa Program Rollover Study
	9	no	NCT04551950	Phase 1	r/m+LA	BA+Ch+CR+TI	Bintrafusp Alfa Combination Therapy in Participants With Cervical Cancer (INTR@PID 046)
	10	no	NCT04246489	Phase 2	r/m	BA	Bintrafusp Alfa Monotherapy in Platinum-Experienced Cervical Cancer
	11	no	JPRN-JapicCTI-205458	Phase 2	r/m+LA	BA+Ch+TI	Safety Study of Bintrafusp alfa in Combination with Other Anti-cancer Therapies in Participants with Locally Advanced or Advanced Cervical Cancer
	12	yes	NCT02517398	Phase 1	r/m+LA	BA	MSB0011359C (M7824) in Metastatic or Locally Advanced Solid Tumors
SHR-1701 PD-L1 and TGF-β	13	yes	NCT03774979	Phase 1	r/m	BA	SHR-1701 in Subjects With Metastatic or Locally Advanced Solid Tumors
	14	no	NCT05179239	Phase 3	r/m	BA+Ch+TI	A Study of SHR-1701 Plus Platinum-containing Chemotherapy With or Without BP102 (Bevacizumab) as First-line Treatment in Cervical Cancer

As the firstly approved anti-PD-1 ICB, there were 33 clinical trials related to Pembrolizumab, including 21 in phase II, 19 in Phase II, and just 1 in phase III. 22 clinical trials of Pembrolizumab began after 2019 (including 2019), and in 2022 the number reached the top with nine trials. There were 11 trials concerned with Nivolumab, all of which were phase I and/or phase II trials, as monotherapy or combined with other agents. Nivolumab is often combined with anti-CTLA-4 antibody ipilimumab, not only for recurrent/metastatic cervical cancer in the classical CheckMate 358 (NCT02488759) but also for locally advanced cervical cancer when combined with chemoradiotherapy. For Cemiplimab (anti-PD-1), a phase III trial in conjunction with vaccine therapy showed its result. HLX 10 is mainly used in combination with chemotherapy, and one trial has obtained its result at present. There are six clinical trials using Sintilimab, which are all phase II (one phase I/II). And only one phase II trial in combination with Anlotinib showed its results. Four clinical trials were related to Balstilimab, among which one had results. But one trial had been withdrawn, and one had been terminated.

The advances of PD-L1 lag behind that of PD-1 in the treatment of advanced cervical cancer. The representative drug Atezolizumab has been studied in 20 clinical trials, which were mainly at the phase II stage, with three trials at the phase III stage.

Bispecific antibodies have continued to thrive in recent years. As the approved bispecific antibody, there are six clinical trials related to AK104, including four phase II trials and two phase III trials. And they all started after 2020. The two phase III trials (one for locally advanced cervical cancer, and the other for recurrent/metastatic cervical cancer) demonstrate the urgent need for improvement in the treatment of both types of cervical cancer. For Bintrafusp Alfa (M7824, anti-PD-L1, and TGF-β), there are seven clinical trials (two phase I, one phase I/II, three phase II, and one phase III). The phase I/II trial of HPV vaccine PRGN-2009 alone or in combination with M7824 in HPV -associated cancers (NCT04432597) had shown its results. Another similar bispecific antibody SHR-1701 (anti-PD-L1 and TGF-β) also showed its preliminary result. And there is an ongoing phase III trial of SHR-1701 combined with platinum-containing chemotherapy as the first-line treatment.

## 4 Discussion

The scientometric analysis in this study demonstrated the fast development of immunotherapies in cervical cancer. The publication and citation numbers of this field have kept growing in recent years. The United States and China were the two countries with the most publications. van der Burg SH was the author with the highest number of publications and citations. Santin AD had the highest co-citations, followed by Tewari KS, and van der Burg SH. There were seven clinical trials on the list of the top 10 references with the highest co-citations, in which three trials were associated with ICBs such as Pembrolizumab and Nivolumab, two trials were about vaccine therapies, one trial was about tumor-infiltrating T cells, and one trial was associated with Bevacizumab. And some necessary discussions were also made, followed by the analysis results. Co-occurrence analysis of keywords reflected the core topic of our study, immunotherapies in cervical cancer caused by HPV. And the timeline view of keywords showed the development and the research hotspots of the field along with time. Burst analysis of keywords also detected the hotspots of this field. And it showed that the research of ICBs such as Nivolumab and Pembrolizumab and their related fields were the main topics in recent years.

**Figure 7:**
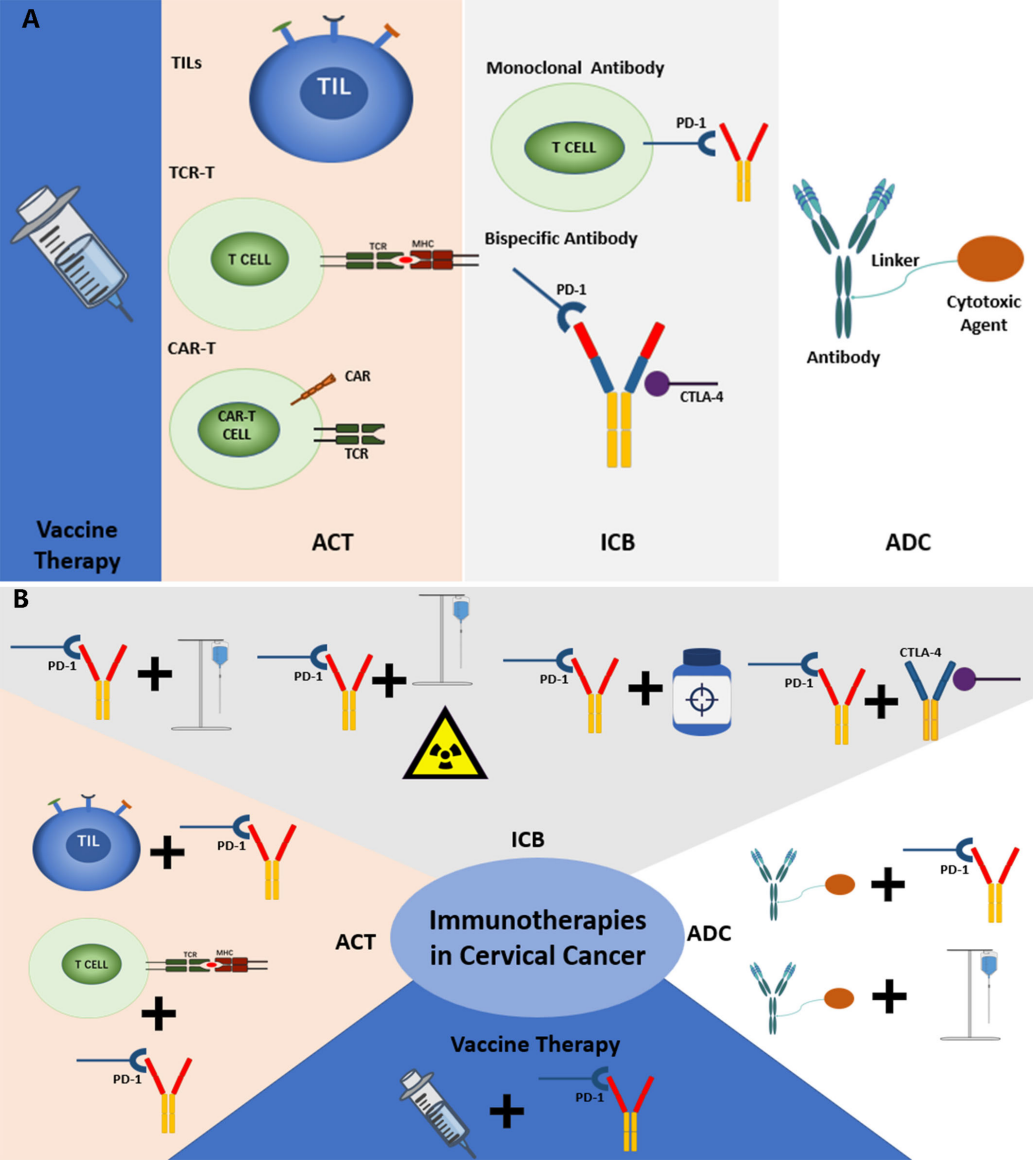
**(A)** The current common immunotherapies in cervical cancer. **(B)** The common combined immunotherapies in cervical cancer.

The analysis of clinical trials in this study also represented some same trends and foci as the scientometric analysis. There are four main kinds of immunotherapies in the current treatment of cervical cancer, including ICBs, Vaccines, ACT, and antibody-drug conjugate (ADC). The distribution of the common treatment modes showed that combined therapies are gradually becoming the new trend, especially ICBs combined with chemotherapy, chemoradiotherapy, and other immunotherapies. Besides, clinical trials of some important immunotherapeutic drugs were also analyzed. Based on the analysis, the common treatment modes and their key drugs were to be further discussed (**Figure 7A, B**).

### 4.1 Vaccine therapy

Therapeutic vaccines used as a care option for cervical cancer patients in clinical trials can date back to 1995 according to our study. During the development of nearly 30 years, patterns of vaccine therapies have changed a lot. Currently, there are four vaccine types in the treatment of cervical cancer, including live vector, protein/peptide, nucleic acid, and cell-based vaccines (39). Most of the vaccines target HPV proteins, mainly E6 and/or E7. And MHC class I and II are necessary for vaccines to stimulate antigen presentation and lead to the responses of CD8+ cytotoxic T cells and CD4+ helper T cells.

Since the effects of single vaccine therapies are usually kind of weak in the treatment of cervical cancer, most clinical trials of vaccine therapies started in recent years are combined with other multiple therapies. And research on the combination of vaccine therapies and ICBs is becoming popular.

VB10.16 plus Atezolizumab. As a DNA vaccine, VB10.16 can deliver the encoded E6 and E7 antigens of HPV16 directedly to the professional antigen-presenting cells and trigger stronger immune responses (40). A phase II trial of VB10.16 in combination with Atezolizumab in patients with advanced or nonresectable HPV 16 positive cervical cancer (NCT04405349) had published its interim analysis results (41). The ORR and DCR (disease control rate) was 20.51% and 64.1%, respectively. Efficacy was observed in patients with PD-L1 positive, PD-L1 negative tumors, and non-inflamed tumors. And the anti-tumor activity seen in the PD-L1 negative population may potentially open up for treatment of a new subset of patients. 10% of patients experienced grade 3 TRAEs (treatment-related adverse events), and no grade 4-5 TRAEs or deaths related to VB10.16 and Atezolizumab were reported (41).

### 4.2 Adoptive cell therapy

T cells are used most in adoptive cell therapies (ACT). And the adoptive T cell therapy is to remove T cells from the hosts, and then expand, modify, or select these T cells for reacting with tumor antigen ex vivo. Then, reinfuse these amplified T cells back into the host’s body. And these T cells will target tumor antigens to promote a stronger immune response and tumor regression (39, 42). There are three main types of ACT in cervical cancer, including tumor-infiltrating lymphocytes (TILs), T-cell receptor (TCR) T cells, and chimeric antigen receptor (CAR) T cells (39). And in our study, the distribution of these ACTs was TILs (n=13, 35.14%), TCR-T (n=9, 24.32%), and CAR-T (n=6, 16.22%). Others include dendric cells (DCs), lymphokine-activated killer cells (LAKs), and cytokine-induced killer cells (CIKs).

#### 4.2.1 TILs

TIL therapy in cervical cancer has gained continuous attention over the past decade. After being separated from a host’s surgically removed tumors, TILs are then expanded ex vivo and reinfused into the host’s body without genetic modifications. These TILs are polyclonal, meaning that they can recognize a range of tumor antigens (43). Currently, there is only one completed clinical trial for TILs to treat cervical cancer (NCT01585428). This was a phase II clinical trial that investigated the application of TILs in patients with metastatic cervical cancer. After lymphocyte-depleting chemotherapy, TILs were chosen to have reactions with HPV E6/E7 antigens and then administered to the patients with aldesleukin (recombinant human interleukin-2) (27). There were three of the nine patients experienced objective tumor responses. And the additional toxicity was limited (27). There are several other ongoing clinical trials using TILs, and four of them had just started in 2022.

LN-145 plus Pembrolizumab. LN-145 is a type of autologous TIL therapy (43). And C-145-04 (NCT03108495) is an ongoing phase II study of LN-145 plus Pembrolizumab as the first-line treatment in patients with recurrent, metastatic, or persistent cervical cancer. There were 14 patients in the analysis (44). The ORR and DCR were 57.1% (95% CI: 28.9-82.3) and 92.9% (95% CI: 66.1-99.8), respectively. And 71.4% of patients with complete or partial responses had ongoing responses at the data cutoff. 85.7% of patients experienced a reduction in tumor burden. Grade 3 or above TEAEs (treatment-emergent adverse events) occurred in 92.9% of patients. Anemia (50.0%), febrile neutropenia (35.7%), thrombocytopenia (35.7%), and neutropenia (28.6%) were the most common grade 3/4 TEAEs (44). Although the combination of LN-145 and Pembrolizumab showed encouraging efficacy with expected safety, more continued investigations are needed.

#### 4.2.2 TCR-T

Different from amplifying tumor-specific T cells that have existed in the host’s body, TCR-T therapy uses modified T cells through genetic engineering, which can express a particular T cell receptor (TCR) to target tumors (45). And these T cells with specific TCR are expanded and then reinfused into the host’s body, to induce a stronger immune response by recognizing particular antigens bound to MHC I/II. It has been studied that the engineered TCR-T cells are capable of identifying HPV E6+ and E7+ tumor cells (45). And there is also a completed phase I/II clinical trial of E6 TCR-T cells combined with lymphocyte depletion and IL2 for patients with metastatic HPV 16-positive cancer that had received prior platinum-based therapy (NCT02280811). The result showed that two of nine subjects in the highest dose cohort experienced objective responses. And one resected tumor tissue demonstrated infiltration by E6 T cells that showed increased expression of PD-1 when compared with E6 T cells in the peripheral blood (26% versus 2%) (45). This indicated the passive role of PD-1/PD-L1 interaction in TCR-T therapy, and TCR-T therapy plus anti-PD-1/PD-L1 therapies may improve the treatment effect (NCT03578406).

#### 4.2.3 CAR-T

CAR-T therapy requires a genetic redirection of T cell specificity by introducing chimeric antigen receptor (CAR), namely a synthetic recognition construction onto the host T cell (46). And this determines the major benefit of CAR-T therapy when compared with TCR-T or TIL therapy, that CAR-T cells have their own recognition structures instead of the need for the intact MHC presentation system. And it is very significant as MHC presentation can be downregulated in the immunosuppressive TME (47). Another difference is that CAR-T therapy uses CD70, GD2, PSMA, Muc1, and Mesothelin as the target instead of HPV antigens (NCT03356795, NCT05420545, NCT05393635, and NCT05518253). However, there is no completed clinical trial using CAR-T therapy in the field of cervical cancer. And CAR-T therapy still has a long way to go before it is approved to apply in solid tumors including cervical cancer.

### 4.3 Immune checkpoint blockades

Since the mechanisms of tumor antigen presentation and tumor microenvironment (TME) are explored more deeply, ICBs and their related fields have been a popular topic in the field of cervical cancer. And until now, ICBs also have made significant progress . There are two types of immune checkpoints, co-stimulatory immune checkpoints such as OX40, ICOS, and 4-1BB (CD137), and co-inhibitory immune checkpoints such as PD1, CTLA-4, and LAG-3 (48, 49). And this determines the two patterns of immune checkpoint blockades. One is agonists on co-stimulatory immune checkpoints, and the other is antagonists on co-inhibitory immune checkpoints (39). Currently, the main progression and successful applications focus on the antagonists against co-inhibitory immune checkpoints, though the agonists on co-stimulatory immune checkpoints are also being explored in several clinical trials for cervical cancer (NCT03241173, NCT03799003, NCT03126110, and NCT03829501).

The mechanism of immune checkpoint blockades is relatively simple. Here, take PD-1/PD-L1 as an example. When the inhibitory immune checkpoint PD-1 expressed by some kinds of immune cells such as CD8+ T cells and (or) its corresponding ligand PD-L1 expressed by tumor cells are blocked, the exhaustion of T cells will be impaired, and the immune system will be enhanced (50–52). Besides the widely researched target such as CTLA-4 and PD-1/PD-L1, some novel co-inhibitory immune checkpoints and their antibodies have also been explored in cervical cancer, such as TIGIT, LAG-3, and TIM-3.

Our study incorporated 221 clinical trials associated with ICBs, and there are multiple treatment patterns for cervical cancer currently.

#### 4.3.1 ICB combined with chemotherapy

Chemotherapy is the fundamental therapy for recurrent or primary metastatic cervical cancer. In the history of chemotherapy for cervical cancer, cisplatin monotherapy and then a combination of cisplatin and paclitaxel were considered the standard therapies in sequence (53). And the addition of Bevacizumab to chemotherapy in the last decade demonstrated a favorable result in the aspect of response rate and overall survival (53). Chemotherapy can not only result in the death of tumor cells, but also make immune cells rupture and then promote the release of tumor-specific antigens, which is beneficial to the immune response. And this special role also provides a possible superior effect on cervical cancer when chemotherapy is combined with immunotherapies. There are 18 clinical trials in our study that use chemotherapies combined with ICBs to treat advanced cervical cancer. Most trials were designed for recurrent or metastatic cervical cancer. And the remaining trials were mainly designed for locally advanced cervical cancer as a neoadjuvant therapy before surgery.

Pembrolizumab plus platinum-based chemotherapy. The phase III clinical trial KEYNOTE-826 (NCT03635567) is the leading one in this treatment pattern. Compared with platinum-based chemotherapy plus placebo with or without Bevacizumab, patients treated by platinum-based chemotherapy plus Pembrolizumab with or without Bevacizumab showed better mPFSs (10.4 months versus 8.2 months; HR=0.65, 95% CI: 0.53-0.79, P<0.001) and mOSs (24.4 months versus 16.5 months; HR=0.67, 95% CI: 0.54-0.84, P<0.001). Through further analysis of subgroups, considerable benefits were gained in the subgroup PD-L1 combined positive score (CPS) of 1 or more, the subgroup PD-L1 CPS of 10 or more, and all the incorporated populations (35). And the higher ORRs were also gained in patients that received combined therapy (65.9% versus 50.8%) (35). These results further supported that Pembrolizumab plus chemotherapy with or without Bevacizumab could be a novel standard care as the first-line for patients with persistent, recurrent, and metastatic cervical cancer (35).

HLX10 plus albumin-bound paclitaxel. A phase II clinical study (NCT04150575) investigated HLX10 (a new anti-PD-1 antibody) combined with albumin and paclitaxel as second-line therapy for advanced cervical cancer or patients with advanced cervical cancer who could not tolerate toxicity (54). In 21 patients with the CPS>=1 (median follow-up duration of 14.6 months), the ORRs and DCRs were 57.1% (95% CI: 34.0-78.2) and 76.2% (95% CI: 52.8-91.8), respectively. The median DoR (duration of response) was not reached, and the mPFS and mOS were 5.7 months and 15.5 months, respectively. The grade 3 or above treatment-emergent adverse events (TEAEs) included decreased neutrophil counts (33.3%), decreased white blood cell count (28.6%), and anemia (19.0%). No TEAEs leading to drug discontinuation were observed. Compared with the former therapies in the second line of treatment such as single albumin-bound paclitaxel (55) or single Pembrolizumab (35), considerable advantages are revealed in the combination therapy of HLX10 and albumin-bound paclitaxel. Besides, a phase III study of HLX10 plus chemotherapy (paclitaxel plus cisplatin and paclitaxel plus carboplatin) as the first-line in patients with advanced cervical cancer was registered in 2021, but it was withdrawn currently (NCT04806945).

Other ongoing phase III clinical trials using this pattern of treatment include NCT03556839 (37), NCT04864782, and NCT03912415.

#### 4.3.2 ICB combined with chemoradiotherapy

The standard care for patients with locally advanced cervical cancer is platinum-based concurrent chemoradiotherapy (CCRT) (56). Compared with neoadjuvant chemotherapy followed by surgery, CCRT showed superior DFS (disease-free survival) besides the similar OS benefit (57, 58). Therefore, the currently preferred combination therapy for locally advanced cervical cancer is chemoradiotherapy plus ICB. There were 26 clinical trials using this treatment pattern in our study. And 92.31% of them were designed for locally advanced cervical cancer. However, the development and progress are not remarkable.

Atezolizumab plus chemoradiotherapy. A phase I/Ib study of Atezolizumab prior to and concurrently given with chemoradiation (CRT) treatment (Arm A, where patients received one dose of Atezolizumab prior to CRT, and two doses during CRT) or concurrently given with CRT (Arm B, where patients received all three doses of Atezolizumab during CRT treatment) for women with node-positive, locally advanced cervical cancer (NRG-GY017) showed its preliminary results (59). In this study, the expansion of TCR clones in peripheral blood and the expansion of tumor-associated T cell clones between baseline and day 21 were observed increased in both Arm A and Arm B. And no significant difference was observed between these two arms (59). Patients with high pre-treatment TCR diversity were more likely to be observed to have complete remission (p= 0.049). And the DFS at 12 months for the entire cohort is 72% (59). Besides, the safety of this combination therapy was also proved.

Another phase I study (NCT01711515) also found that Ipilimumab following definitive chemoradiation can improve immune activation (60). Currently, the majority of the clinical trials of this kind of regimen are being actively conducted, such as a phase II study of Dostarlimab as maintenance therapy after chemoradiation (61), and a phase III study of Durvalumab with the following concurrent chemoradiotherapy (38).

#### 4.3.3 ICB combined with targeted therapy

Targeted therapies have countered lots of difficulties in the treatment of advanced cervical cancer, making it necessary and urgent to develop other novel therapies. And in this situation, immunotherapy has been regarded as a new hope, especially when it is combined with other therapies. Currently, the combined regimen that use both ICBs and targeted therapies such as small-molecule inhibitors is widely being studied in clinical trials, some of which has represented promising results.

Sintilimab plus Anlotinib. Anlotinib is a multi-targeting tyrosine kinase inhibitor (TKI) with targets on VEGFR (vascular endothelial growth factor receptor), FGFR (fibroblast growth factor receptor), PDGFR (platelet-derived growth factor receptor), and c-kit (62). And the result of a phase II clinical trial of Sintilimab plus Anlotinib as the second line or later therapy for PD-L1 positive recurrent or metastatic cervical cancer was published recently. The ORR and DCR were 54.8% (95% CI, 38.7-70.2) and 88.1% (95% CI: 74.4-96.0), respectively. And in efficacy-evaluable patients, the ORR and DCR were 59.0% (95% CI, 42.1 to 74.4) and 94.9% (95% CI, 82.7 to 99.4). The mPFS was 9.4 months (95% CI: 8.0-14.6), and the mOS was not reached. 85.8% of the patients experienced TRAEs, and these TRAEs were consistent with the reported ones for Sintilimab and Anlotinib. 16.7% of patients reported grade 3 or above TRAEs. Besides, a higher ORR in patients with altered PIK3CA, PI3K-AKT signaling, or KMT2D, and a shorter PFS in patients with altered STK11 and/or JAK2 were observed (63). Overall, this study of Sintilimab plus Anlotinib showed promising efficacy and acceptable safety, but a phase III trial with a larger is needed to prove the more precise efficacy.

Nivolumab plus Lucitanib. Lucitanib is an antiangiogenic TKI that selectively inhibits VEGFR 1−3, FGFR 1−3, and PDGFRα/β (64). A phase II study to test the efficacy and safety of Nivolumab (a PD-1 inhibitor) plus Lucitanib to treat patients with advanced gynecologic malignancies (including persistent or recurrent cervical cancer) (NCT04042116) showed its results in 2022 ASCO (American Society of Clinical Oncology) (65). The confirmed ORR and DCR were 26.1% (95%CI: 14.3-41.1) and 47.8% (95%CI: 32.9-63.1), respectively. The duration of confirmed responses ranged from 1.9+ to 13.1 months. The mPFS was 5.5 months (95%CI: 3.2–10.9). In the aspect of safety, 92.7% of the patients with advanced gynecologic malignancies experienced TRAEs. And 44.4% of patients reported grade 3 or above TRAEs (65).

Currently, there are 25 ongoing phase II (phase I/II) clinical trials using this treatment pattern in patients with advanced cervical cancer in our study. And in these trials, targeted inhibitions especially multi-targeting tyrosine kinase inhibitors such as Anlotinib, Famitinib, Lenvatinib, and Cabozantinib are widely applied.

#### 4.3.4 ICBs in combination

Since the effect of monotherapy for ICBs is often limited, several ICBs in combination are expected to enhance anti-tumor activity (28).

Balstilimab and Zalifrelimab. Balstilimab is an anti-PD-1 monoclonal antibody, and Zalifrelimab is an anti-CTLA-4 monoclonal antibody. A phase II study of Balstilimab and Zalifrelimab in combination as the second-line treatment for advanced cervical cancer had shown its results (66). The confirmed ORR was 25.6% (95% CI: 18.8%-33.9%), and the median DoR was not reached (86.5%, 75.5%, and 64.2% at 6, 9, and 12 months, respectively). And the ORRs were 32.8% and 9.1% in PD-L1 positive and PD-L1 negative patients, respectively. The overall DCR was 52% (95% CI: 43.3-60.6). The most common immune-related adverse events were hypothyroidism (14.2%) and hyperthyroidism (7.1%) (66). Besides, there is also a randomized phase II study of Balstilimab alone or combined with Zalifrelimab in patients with recurrent or metastatic cervical cancer (NCT03894215) ongoing currently (67). But this clinical trial was aborted recently in China.

#### 4.3.5 Bispecific antibodies

Depending on the rapid development of bispecific antibody technologies, there are more and more novel bispecific antibodies targeting two immune checkpoints or one immune checkpoint and one other molecule such as TGF-β. The better anti-tumor efficacy is obtained in preclinical experiments when using bispecific antibodies instead of the mAbs and their combination (68, 69). In June 2022 Cadonilimab (a bispecific antibody targeting PD-1 and CTLA-4) was approved in China to treat patients with relapsed or metastatic cervical cancer (17). And a few other bispecific antibodies are also being actively explored in clinical trials, some of which have indicated excellent results.

Cadonilimab. Cadonilimab (AK104) is a PD-1/CTLA-4 bispecific antibody that had been approved in China to treat patients with relapsed or metastatic cervical cancer who have progressed on or after platinum-based chemotherapy (17). And for the first-line of treatment, a phase III study of AK104 combined with standard therapy (platinum-based chemotherapy, with or without Bevacizumab) to treat persistent, recurrent, or metastatic cervical cancer (NCT04868708) showed remarkable results (49). Patients were assigned to three cohorts (1A-15/2A-10: AK104 15/10 mg/kg plus Paclitaxel 175mg/m^2^ plus Cisplatin 50 mg/m^2^/Carboplatin AUC 5, q3w; 3B-10: AK104 10 mg/kg plus Paclitaxel 175 mg/m^2^ plus Cisplatin 50 mg/m^2^/Carboplatin AUC 5 plus Bevacizumab 15 mg/kg, q3w) (70). In the three cohorts (A-15, A-10, and B-10), the ORRs were 66.7%, 68.8%, and 92.3%, and the DCRs were 100%, 93.8%, and 100%, respectively. The response to the treatment was favorable regardless of CPS. And the tolerance of the combined therapies was controllable. Grade 3 or above TRAEs occurred in 60.0% of patients. The most common TRAEs were anemia (66.7%), white blood cell count decreased (57.8%). TRSAE occurred in 44.4% of patients. Grade 3 or more irAE occurred in 15.6% of patients. One death occurred in cohort B-10 which was judged as Bevacizumab-related (70).

There are some other ongoing trials of AK104, such as AK104 as a neoadjuvant treatment for IB2-IIA2 cervical cancer (NCT05227651), and a phase II study of AK104 in patients with recurrent or metastatic high-grade neuroendocrine cervical cancer (NCT05063916).

GEN1046. GEN1046 is a PD-L1/4-1BB bispecific antibody that has a special mechanism. It can exhibit the conditional 4-1BB agonist activity that relies on the cross-linking with PD-L1. But when playing the role of the PD-L1 inhibitor, it is independent of 4-1BB binding (71, 72). *Via* this special mechanism, GEN1046 represents the enhancement of immune function and anti-tumor activity in both preclinical research and phase I trial (71).

Besides 4-1BB, other co-stimulatory checkpoints such as ICOS, OX40, and GITR are also being explored in clinical trials. However, the development of their monoclonal antibodies is not going as well as co-inhibitory immune checkpoints. And one of the key points is their potential toxicity (20, 71, 73). Now it is promising for agonistic antibodies of these co-stimulatory molecules to shine again through bispecific antibodies.

M7824. M7824 (Bintrafusp alfa) is a bifunctional protein composed of an anti-PD-L1 antibody that is fused to the extracellular domain of TGF-β receptor II (functioning as a TGF-β trap) (74). The TGF-β/Smad pathway is an important contributor to the stronger invasion, metastasis, and immunosuppression of cervical cancer, which made it become a popular target in this kind of bispecific antibodies (74, 75). In the combined analysis of a phase I study of M7824 in metastatic or locally advanced solid tumors (NCT02517398) and a phase II study of M7824 in HPV-associated malignancies (NCT03427411), the manageable safety profiles and clinical activities were shown to support the feasibility of M7824 as the second line treatment (76). The ORR was 28.2% (95% CI: 15.0-44.9), and the median DoR was 11.7 months (range: 1.4-41.2 months). Responses occurred regardless of tumor histology, prior Bevacizumab, and radiation treatment. The mOS was 13.4 months (95% CI: 5.5 to NR), and the 24-month OS rate was 33.2% (76). Grade 3 or above TRAEs occurred in 20.5% of patients. And no treatment-related deaths occurred.

There are two other ongoing clinical trials about M7824. One is to assess the efficacy and safety of M7824 monotherapy in patients with advanced, unresectable cervical cancer who have disease progression during or after platinum-containing chemotherapy (NCT04246489). The other is to evaluate the safety and tolerability of M7824 in combination with other therapies (cisplatin/carboplatin plus paclitaxel with or without Bevacizumab, cisplatin plus radiotherapy) as the first-line in locally advanced cervical cancer (NCT04551950).

SHR-1701. SHR-1701 is a similar bifunctional fusion protein to M7824. In a phase I study of SHR-1701 in 32 recurrent or metastatic cervical cancer, the ORR and DCR were 15.6% (95% CI: 5.3-32.8) and 50.0% (95% CI: 31.9-68.1), respectively. The 6-month duration of response rate was 80.0% (95% CI: 20.4–96.9). There was no difference in ORR between patients with CPS>=1 and CPS<1. And patients with high phosphorylated SMAD2 levels in immune cells or tumor cells had numerically higher ORR (77). The mPFS and immune-modified RECIST mPFS were 2.7 months (95% CI: 1.4–4.1) and 4.1 months (95% CI: 1.6–4.3), respectively. The overall survival rate at 12 months was 54.6% (95% CI: 31.8–72.7) (77). Grade 3 or above TRAEs were reported in 11 (34.4%) patients. No treatment-related deaths occurred (77).

In 2022, a phase III study of SHR-1701 plus paclitaxel and cisplatin/carboplatin with or without Bevacizumab as the first-line treatment in persistent, recurrent, or metastatic cervical cancer had started (NCT05179239).

Besides, the quick development of antibody technologies has brought new chances to block or bind more molecules, such as tri-specific antibodies and tetra-specific antibodies. And the tri-specific antibodies have been proven to induce stronger T cell activation and anti-tumor effects *in vitro* and *in vivo* (78, 79). Based on the specific molecular characteristics of cervical cancer, it is hopeful that personalized cocktail therapy can be formed through the combination of multiple antibodies.

### 4.4 Antibody-drug conjugate

ADCs have a unique construction that consists of an antibody (to recognize a particular cellular antigen), a cytotoxic molecule (bound to the antibody), and a linker (to hold the two parts together) (80). ADCs are designated to improve the efficacy of chemotherapy at the condition of limiting toxicity to the whole body. Based on the highly specific antigen-antibody interaction model, the ADCs can directly deliver the cytotoxic molecule into the targeted cancer cells, which can induce a stronger killing effect (81). Currently, there have been two main kinds of cellular antigens used as feasible targets in cervical cancer for ADCs, TF (tissue factor) and Trop2 (anti-human trophoblast cell-surface marker) (80, 82).

Tisotumab Vedotin. TF is highly expressed in solid tumors such as cervical, and it is not or lowly expressed in normal cervical tissues. This supports TF to act as an ideal target for ADCs. The anti-TF ADC Tisotumab Vedotin is the leading ADC in the field of cervical cancer. Tisotumab Vedotin showed good results both as a single agent and in combination with other therapies. In a phase II study of Tisotumab Vedotin as the second-line treatment in patients with recurrent or metastatic cervical cancer, the ORR was 24% (95% CI: 16–33; 7% CR, and 17% PR). The median DoR, PFS, and OS were 8.3 months (95% CI: 4.2–NR), 4.2 months (95% CI: 3.0–4.4), and 12.1 months (95% CI: 9.6–13.9), respectively. Grade 3 or worse TRAEs were reported in 28% of patients, including neutropenia (3%), fatigue (2%), ulcerative keratitis (2%), and peripheral neuropathies (2% each with sensory, motor, sensorimotor, and neuropathy peripheral) (83). A phase III trial of Tisotumab Vedotin combined with Pembrolizumab or Carboplatin in the first-line treatment in patients with recurrent/metastatic cervical cancer. In the first-line treatment of Tisotumab Vedotin plus Pembrolizumab (1L TV + Pembro) cohort, the confirmed ORR, the median time of response, and the mDoR were 40.6% (95% CI: 23.7-59.4), 1.4 months (95% CI: 1.2-2.8), and not reached (2.8-21.9+ months), respectively. In the second/third line treatment of Tisotumab Vedotin plus Pembrolizumab (2/3L TV + Pembro) cohort, the confirmed ORR, the median time of response, and the mDoR were 38.2% (95% CI: 22.2-56.4), 1.4 months (95% CI: 1.3-5.8), and 14.0 months (2.8-NR), respectively. In the first-line treatment of Tisotumab Vedotin plus Carboplatin (1L TV + Carbo) cohort, the confirmed ORR, the median time of response, and the mDoR were 54.5% (95% CI: 36.4-71.9), 1.4 months (95% CI: 1.1-4.4), and 8.6 months (4.2-11.5), respectively. The TRAEs were mostly grade 1 or 2, and mainly included ocular, peripheral neuropathy, and bleeding events. The observed safety is generally consistent with that of each known agent. A single grade 5 event with 1L TV + Pembro occurred (due to disseminated intravascular coagulation). And the immune-mediated AEs observed with TV + Pembro were in keeping with the known safety profile of checkpoint inhibitors.

### 4.5 Where are we, and where are we going?

Immune-based regimens have been the standard recommendation in the treatment guidelines for more and more solid tumors, such as non-small cell lung cancer, melanoma, and gastric cancer. And for advanced cervical cancer, we have also made significant progress, and the immune-based treatment era is also closer and closer. However, we still lack the support of a large number of multicentered, randomized, controlled phase III clinical trials.

From **Table 1** to **Table 3**, it could be easily found that most of the phase III trials are still in recruiting status. And there were only a few clinical trials with published results (**Table 4**), including five trials in the first line (one phase III and four phase I or II), and ten trials in the second line or above (one phase III and nine phase I or II). Meanwhile, most of these trials used ORR as the primary outcome with a relatively small enrollment size.

**Table 4:** The clinical trials for recurrent/metastatic cervical cancer with published results.

Drugs	No.	Trial ID	Phase	Size	Primary outcome	Title
Pembrolizumab vs. placebo	1	NCT03635567	Phase 3	617	PFS:10.4m vs.8.2m; OS(24m):50.4% vs.40.4%	Pembrolizumab for Persistent, Recurrent, or Metastatic Cervical Cancer (35)
Pembrolizumab	2	NCT02628067	Phase 2	98	ORR:12.2% (ITT) vs. 14.6% (PD-L1+)	Efficacy and Safety of Pembrolizumab in Previously Treated Advanced Cervical Cancer: Results From the Phase II KEYNOTE-158 Study (24)
Pembrolizumab plus GX-188E	3	NCT03444376	Phase 2	26	ORR: 31.7% (ALL) vs.25.0% (PD-L1-)	Pembrolizumab plus GX-188E therapeutic DNA vaccine in patients with HPV-16-positive or HPV-18-positive advanced cervical cancer: interim results of a single-arm, phase 2 trial (84)
Nivolumab	4	NCT02488759	Phase 1|2	24	ORR: 26.3% (Cervical) vs. 20.0% (Virginal Vulvar)	Safety and Efficacy of Nivolumab Monotherapy in Recurrent or Metastatic Cervical, Vaginal, or Vulvar Carcinoma: Results From the Phase I/II CheckMate 358 Trial (28)
Atezolizumab plus Bevacizumab	5	NCT02921269	Phase 2	10	ORR: 0%; DCR: 60%; PFS: 2.9m; OS: 8.9m	Phase II study of atezolizumab in combination with bevacizumab in patients with advanced cervical cancer (85)
Cemiplimab vs. chemotherapy	6	NCT03257267	Phase 3	608	OS: 12m vs. 8.5m, HR=0.56; ORR: 18%	Survival with Cemiplimab in Recurrent Cervical Cancer (36)
Bintrafuspalfa	7	NCT04551950	Phase 1	25	ORR: 62.5% vs. 33.3% vs. 62.5%	Bintrafusp Alfa Combination Therapy in Participants With Cervical Cancer (INTR@PID 046) (86)
QL1706	8	NCT05171790	Phase 1	53	ORR:28%; PFS:4.2m	Efficacy and safety of QL1706, a novel dual immune checkpoint blockade containing a mixture of anti-PD1 IgG4 and anti-CTLA4 IgG1 antibodies, for advanced cervical cancer: Cohort data from a phase 1b trial (87).
Serplulimab vs. chemotherapy	9	NCT04150575	Phase 2	21	ORR: 57.1%; PFS (12m): 48.2%; OS (12m): 71.1%	Efficacy and safety of serplulimab (an anti-PD-1 antibody) combined with albumin-bound paclitaxel in patients with advanced cervical cancer who have progressive disease or intolerable toxicity after first-line standard chemotherapy (074) (88)
Cadonilimab	10	NCT03852251	Phase 2	111	ORR: 33% (ITT), 43.8% (PD-L1+); PFS: 3.75m (ITT), 6.34m (PD-L1+)	Efficacy and safety of cadonilimab, an anti-PD1/CTLA4 bi-specific antibody, in previously treated recurrent or metastatic (R/M) cervical cancer: A multicenter, openlabel, single-arm, phase II trial (89)
Cadonilimab	11	NCT04380805	Phase 2	30	ORR: 47.6%	Efficacy and Safety of Cadonilimab, An Anti-PD-1/CTLA4 Bi-specific Antibody, in Previously Treated Recurrent or Metastatic (R/M) Cervical Cancer: A Multicenter, Open-label, Single-arm, Phase II Trial (89)
Cadonilimab+Chemotherapy± Bevacizumab	12	NCT04868708	Phase 2	45	ORR: 66.7% (A-15) vs. 68.8% (A-10) vs. 92.3% (B-10)	A study of AK104 (an anti-PD1 and anti-CTLA4 bispecific antibody) combined with standard therapy for the first-line treatment of persistent, recurrent, or metastatic cervical cancer (R/M CC) (70)
Socazolimab	13	NCT03676959	Phase 1	104	ORR: 15.4% (ITT) vs. 16.7% (PD-L1+)	Efficacy and Safety of the Anti-PD-L1 mAb Socazolimab for Recurrent or Metastatic Cervical Cancer: a Phase I Dose-Escalation and Expansion Study (90)

ITT, intend to treat; HR, hazard ratio.

Nonetheless, the published results of some clinical trials are also worthy of our excitement and expectation. Most immune-based regimens represent higher ORRs than the standard treatments or immunotherapy alone, both in the first line and the second line or above. And the ORRs in PD-L1 positive subgroup is usually higher than in PD-L1 negative subgroup (24, 84, 89, 90). Besides, the most promising explorations of ADC-based and ICB (especially bispecific antibody)-based regimens are on the way as discussed above, which need to be tested further in clinical trials.

## Data availability statement

The original contributions presented in the study are included in the article/**Supplementary Material**. Further inquiries can be directed to the corresponding authors.

## Author contributions

HW, and ZL conceived the study and performed critical revision of the manuscript. YX designed the study, performed statistical analyses, and drafted the manuscript. FY, YD, HG, MH, YZ, RG, LY, JC, and DM designed the study and wrote the manuscript. YX, FY, YD, and DM performed the article retrieval and data interpretation. All authors read and approved the final manuscript.
