# Supplementary material for: Immunotherapy in cervical cancer: From the view of scientometric analysis and clinical trials
Source: Front Immunol. 2023 Feb 3;14:1094437. doi: 10.3389/fimmu.2023.1094437 (PMC9935705; doi:10.3389/fimmu.2023.1094437)
Supplement: Supplementary file 2 [file Table_1.docx]

**Table S1 | The important anti-PD-1/PD-L1 drugs and their correspondingly selected clinical trials.**

| Drugs | No. | Has result | Trial ID | Phase | Cancer Stage | Treatment Mode | Title |
| --- | --- | --- | --- | --- | --- | --- | --- |
| Pembrolizumab  PD-1 | 1 | yes | NCT03444376 | Phase 1\|Phase 2 | r/m+LA | ICB+V | The Combination of GX-188E Vaccination and Pembrolizumab in Patients With HPV 16 and/or 18+ Advanced Cervical Cancer |
|  | 2 | yes | NCT03192059 | Phase 2 | r/m | ICB+Ra | Study of Pembrolizumab, Radiation and Immune Modulatory Cocktail in Cervical/Uterine Cancer |
|  | 3 | yes | NCT02628067 | Phase 2 | r/m+LA | ICB | Study of Pembrolizumab (MK-3475) in Participants With Advanced Solid Tumors (MK-3475-158/KEYNOTE-158) |
|  | 4 | yes | NCT03635567 (EUCTR2018-001440-53-DE) | Phase 3 | r/m | ICB+Ch | Efficacy and Safety Study of First-line Treatment With Pembrolizumab (MK-3475) Plus Chemotherapy Versus Placebo Plus Chemotherapy in Women With Persistent, Recurrent, or Metastatic Cervical Cancer (MK-3475-826/KEYNOTE-826) |
|  | 5 | no | NCT04865887 | Phase 2 | r/m+LA | ICB+TI | Pembrolizumab and Lenvatinib in Advanced Cervical Cancer |
|  | 6 | no | NCT04641728 (JPRN-jRCT2031210096) | Phase 2 | r/m | ICB+TI | Pembrolizumab Plus Olaparib in Patients With Recurrent Cervical Cancer |
|  | 7 | no | NCT04483544 | Phase 2 | r/m | ICB+TI | Pembrolizumab and Olaparib in Cervical Cancer Patients |
|  | 8 | no | NCT04238988 | Phase 2 | LA | ICB+Ch | Carboplatin-Paclitaxel-Pembrolizumab in Neoadjuvant Treatment of Locally Advanced Cervical Cancer |
|  | 9 | no | NCT04221945 | Phase 2 | LA | ICB+CR | Study of Chemoradiotherapy With or Without Pembrolizumab (MK-3475) For The Treatment of Locally Advanced Cervical Cancer (MK-3475-A18/KEYNOTE-A18/ENGOT-cx11/GOG-3047) |
|  | 10 | no | NCT03367871 | Phase 2 | r/m | ICB+Ch+TI | Combination Pembrolizumab, Chemotherapy and Bevacizumab in Patients With Cervical Cancer |
| Cemiplimab  PD-1 | 11 | no | NCT04646005 | Phase 2 | r/m | ICB+V | Cemiplimab and ISA101b Vaccine in Adult Participants With Recurrent/Metastatic Human Papillomavirus (HPV)16 Cervical Cancer Who Have Experienced Disease Progression After First Line Chemotherapy |
|  | 12 | yes | NCT03257267 | Phase 3 | r/m | ICB | Study of Cemiplimab in Adults With Cervical Cancer |
| Nivolumab  PD-1 | 13 | yes | NCT04042116 | Phase 1\|Phase 2 | r/m | ICB+TI | A Study to Evaluate Lucitanib in Combination With Nivolumab in Patients With a Solid Tumor |
|  | 14 | no | NCT03298893 | Phase 1\|Phase 2 | LA | ICB+CR | Nivolumab in Association With Radiotherapy and Cisplatin in Locally Advanced Cervical Cancers Followed by Adjuvant Nivolumab for up to 6 Months |
|  | 15 | yes | NCT02488759 | Phase 1\|Phase 2 | r/m+LA | ICBs | Non-Comparative, Open-Label, Multiple Cohort, Phase 1/2 Study of Nivolumab Monotherapy and Nivolumab Combination Therapy in Subjects With Virus-Positive and Virus-Negative Solid Tumors |
|  | 16 | no | NCT05504642 | Phase 2 | LA | ICBs+CR | Chemo-radio-immunotherapy With Nivolumab and Ipilimumab Treatment in Locally Advanced Cervical Cancer Patients |
|  | 17 | no | NCT05492123 | Phase 2 | LA | ICBs+CR | Nivolumab-ipilimumab and Chemoradiation for Cervical Cancer |
|  | 18 | yes | NCT02257528 | Phase 2 | r/m | ICB | Nivolumab in Treating Patients With Persistent, Recurrent, or Metastatic Cervical Cancer |
| HLX-10  PD-1 | 19 | no | NCT05444374 | Phase 2 | r/m | ICB+TI | A Phase II Study of Serplulimab Plus Bevacizumab in Combination With Chemotherapy in 1L Treatment of Untreated Recurrent or Metastatic Cervical Cancer |
|  | 20 | yes | NCT04150575 | Phase 2 | r/m | ICB+Ch | A Clinical Study to Evaluate Efficacy and Safety of HLX10 Combined With Albumin-Bound Paclitaxel in Patients With Advanced Cervical Cancer Who Have Progressive Disease or Intolerable Toxicity After First-Line Standard Chemotherapy |
| Sintilimab  PD-1 | 21 | no | NCT05105672 | Phase 2 | LA | ICB+CR | A Study of Sintilimab Combined With Concurrent Chemoradiation Therapy in Locally Advanced Cervical Cancer |
|  | 22 | no | NCT04590599 | Phase 2 | r/m+LA | ICBs | A Clinical Study of Sintilimab Independently or in Combination With IBI310 (Anti-CTLA4) in Second-Line Cervical Cancer |
|  | 23 | no | ChiCTR2100049267 | Phase 2 | r/m | ICB+Ch+TI | Phase II clinical study of albumin-paclitaxel + cisplatin/carboplatin combined with bevacizumab and sintilimab in the treatment of stage IVb, recurrent, persistent cervical cancer and endometrial cancer |
|  | 24 | no | ChiCTR1900028540 | Phase 2 | r/m | ICB+Ch | Efficacy and Safety Study for Paclitaxel plus Cisplatin Chemotherapy with Sintilimab in Women with Persistent, Recurent, or Metastatic Cervical Cancer |
|  | 25 | yes | ChiCTR1900023015 | Phase 2 | r/m | ICB+TI | Single-center, one-arm, prospective phase II clinical study of the efficacy and safety of sintilimab combined with anlotinib in the treatment of persistent, recurrent, and metastatic cervical cancer |
| Balstilimab  PD-1 | 26 | no | NCT05033132 | Phase 2 | r/m+LA | ICBs | A Phase II Study of Balstilimab Independently or in Combination With Zalifrelimab in Advanced Cervical Cancer |
|  | 27 | no | NCT03894215 | Phase 2 | r/m+LA | ICBs | RaPiDS- A Phase 2 Study of Anti-PD-1 Independently or in Combination With Anti-CTLA-4 in Second-Line Cervical Cancer |
|  | 28 | yes | NCT03495882 | Phase 1\|Phase 2 | r/m+LA | ICBs | Subjects With Metastatic or Locally Advanced Solid Tumors, and Expansion Into Select Solid Tumors (Cervical) |
| Atezolizumab  PD-L1 | 29 | yes | NCT03738228 | Phase 1 | LA | ICB+CR | Atezolizumab Before and/or With Chemoradiotherapy in Immune System Activation in Patients With Node Positive Stage IB2, II, IIIB, or IVA Cervical Cancer |
|  | 30 | yes | NCT04405349 | Phase 2 | r/m | ICB+V | Investigating the Combination of VB10.16 and Atezolizumab in Patients With HPV 16-positive Cervical Cancer |
|  | 31 | no | NCT03614949 | Phase 2 | r/m | ICB+Ra | SBRT and Atezolizumab in the Management of Recurrent, Persistent, or Metastatic Cervical Cancer |
|  | 32 | no | NCT03612791 | Phase 2 | LA | ICB+CR | Randomized Phase II Trial Assessing the Inhibitor of Programmed Cell Death Ligand 1 (PD-L1) Immune Checkpoint Atezolizumab in Locally Advanced Cervical Cancer |
|  | 33 | yes | NCT02921269 | Phase 2 | r/m | ICB+TI | Atezolizumab and Bevacizumab in Treating Patients With Recurrent, Persistent, or Metastatic Cervical Cancer |
|  | 34 | no | EUCTR2017-003622-33-FR | Phase 2 | LA | ICB+CR | Randomized Phase II Trial Assessing the Inhibitor of Programmed Cell Death Ligand 1 (PD-L1) Immune Checkpoint Atezolizumab in Locally Advanced Cervical Cancer - AtezoLACC |
|  | 35 | no | NCT04300647 | Phase 2\|Phase 3 | r/m | ICBs | A Study of Tiragolumab Plus Atezolizumab and Atezolizumab Monotherapy in Participants With 3636Metastatic and/or Recurrent PD-L1-Positive Cervical Cancer |
|  | 36 | no | NCT03946358 | Phase 3 | r/m+LA | ICB+V | Combination of UCPVax Vaccine and Atezolizumab for the Treatment of Human Papillomavirus Positive Cancers (VolATIL) |
|  | 37 | no | NCT03556839 | Phase 3 | r/m | ICB+Ch+TI | Platinum Chemotherapy Plus Paclitaxel With Bevacizumab and Atezolizumab in Metastatic Carcinoma of the Cervix |
